# Structure of the Yeast Cell Wall Integrity Sensor Wsc1 Reveals an Essential Role of Surface-Exposed Aromatic Clusters

**DOI:** 10.3390/jof8040379

**Published:** 2022-04-08

**Authors:** Philipp Schöppner, Anne Pia Lutz, Bernard Johannes Lutterbach, Stefan Brückner, Lars-Oliver Essen, Hans-Ulrich Mösch

**Affiliations:** 1 Department of Genetics, Philipps-Universität, Karl-von-Frisch-Strasse 8, 35043 Marburg, Germany; schoepp1@staff.uni-marburg.de (P.S.); anne.lutz@hotmail.de (A.P.L.); bernard.lutterbach@gmx.net (B.J.L.); brueckns@staff.uni-marburg.de (S.B.); 2 Department of Biochemistry, Philipps-Universität, Hans-Meerwein-Strasse 4, 35043 Marburg, Germany

**Keywords:** fungal cell wall, *Saccharomyces cerevisiae*, membrane sensor, cysteine-rich domain, PAN domain, aromatic clusters

## Abstract

In the yeast *Saccharomyces cerevisiae* and other ascomycetes, the maintenance of cell wall integrity is governed by a family of plasma-membrane spanning sensors that include the Wsc-type proteins. These cell wall proteins apparently sense stress-induced mechanical forces at the cell surface and target the cell wall integrity (CWI) signaling pathway, but the structural base for their sensor function is yet unknown. Here, we solved a high-resolution crystal structure of the extracellular cysteine-rich domain (CRD) of yeast Wsc1, which shows the characteristic PAN/Apple domain fold with two of the four Wsc1 disulfide bridges being conserved in other PAN domain cores. Given the general function of PAN domains in mediating protein–protein and protein–carbohydrate interactions, this finding underpins the importance of Wsc domains in conferring sensing and localization functions. Our Wsc1 CRD structure reveals an unusually high number of surface-exposed aromatic residues that are conserved in other fungal CRDs, and can be arranged into three solvent-exposed clusters. Mutational analysis demonstrates that two of the aromatic clusters are required for conferring *S. cerevisiae* Wsc1-dependent resistance to the glucan synthase inhibitor caspofungin, and the chitin-binding agents Congo red and Calcofluor white. These findings suggest an essential role of surface-exposed aromatic clusters in fungal Wsc-type sensors that might include an involvement in stress-induced sensor-clustering required to elicit appropriate cellular responses via the downstream CWI pathway.

## 1. Introduction

The fungal cell wall is a highly specialized and dynamic cellular compartment that plays a key role in the maintenance of cellular integrity and protection of cells against physical stress or mechanical damage [1,2]. Genome-wide studies employing the budding yeast *Saccharomyces cerevisiae* and other fungi have revealed that approximately 20% of fungal genomes are involved in cell wall biosynthesis, function, and regulation [3,4,5]. Comprehensive studies in *S. cerevisiae* have shown that sensing of structural alterations within the cell wall during cell growth, and in response to chemical or mechanical stress, is essential for the viability of fungal cells [6]. These studies have also provided detailed insights into the signaling systems that confer appropriate physiological responses to counteract cell wall stress, such as the treatment of fungal cells with cell-wall-perturbing agents [7,8,9,10,11,12,13]. Together, the sensors and signaling components of this system are named the cell wall integrity (CWI) pathway [6], which can be found not only in *S. cerevisiae*, but, in homologous form, also in other fungi [14]. At its core, the CWI pathway comprises a small number of membrane-spanning sensors, protein kinase C (PKC), a mitogen-activated protein kinase (MAPK) cascade, and a number of nuclear transcription factors that confer expression of cell wall biogenesis genes [15,16].

**Figure 1:**
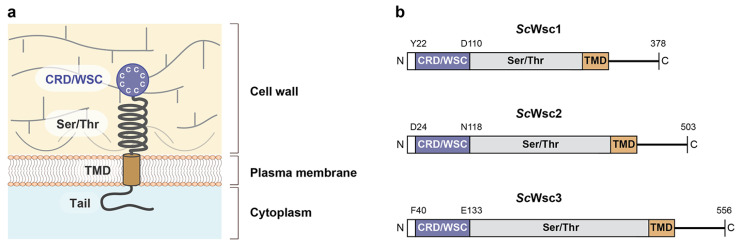
Structural model and architecture of fungal WSC family sensors. (**a**) Model showing the topology and proposed functional domains of WSC domain sensors, including the N-terminal cysteine-rich domain (CRD) or WSC domain, the glycosylated serine/threonine-rich domain (Ser/Thr), the transmembrane domain (TMD), and the C-terminal cytoplasmic tail [17]. (**b**) Domain architecture of the *S. cerevisiae* Wsc family mechanosensors Wsc1 (*Sc*Wsc1, UniProt entry P54867), Wsc2 (*Sc*Wsc2, UniProt entry P53832), and Wsc3 (*Sc*Wsc3, UniProt entry Q12215). Residues corresponding to the borders of the CRD/WSC domains and the C-terminal ends are indicated.

In *S. cerevisiae*, five plasma membrane-spanning proteins have been identified as sensors of the CWI pathway [13,17]. Three of these sensors (*Sc*Wsc1, *Sc*Wsc2, and *Sc*Wsc3) are characterized by an N-terminal, extracellular cysteine-rich domain (CRD), or WSC domain (PFAM entry PF01822; INTERPRO entry: IPR002889), which is followed by an extracellular, glycosylated serine/threonine-rich region (STR), a single transmembrane domain (TMD), and a C-terminal cytoplasmic tail (Figure 1). The best studied variant is *Sc*Wsc1, which has been characterized by genetic, biophysical, and structural methods. Genetic studies have shown that *Sc*Wsc1 is required to confer resistance against numerous types of cell wall stress, including treatment of cells with the β-1,3-glucan synthase inhibitor caspofungin, the chitin-binding agents Congo red and Calcofluor white, or caffeine [12,18]. Further studies have demonstrated that *Sc*Wsc1 function requires the presence of the N-terminal CRD [19], which can also be inactivated by mutation of its eight cysteine residues that are a hallmark of this highly conserved domain [20]. Experiments employing atomic-force microscopy (AFM) further indicate that *Sc*Wsc1 sensors form nanosprings, and act as mechanosensors [21], which can be localized in specific microdomains within the plasma membrane [22]. These studies also suggest that the CRD of *Sc*Wsc proteins is required for sensor clustering observed in response to cell wall stress [20]. Purification of *Sc*Wsc1 from yeast cells in lipid nanoparticles and transmission electron microscopy have recently provided the first broad three-dimensional model, which confirms the proposed tripartite organization of the sensor [23]. These studies suggest that *Sc*Wsc proteins may act as mechanosensors that detect structural alterations of the cell wall by their extracellular CRD and STR domains, and, upon stress, undergo extensive conformational changes, which are transferred to the cytoplasmic tail, and trigger activation of downstream signaling components of the CWI pathway [17,23]. In addition, Wsc1 may have structural functions at the yeast cell wall independently of the CWI pathway.

Although the CRD of yeast Wsc proteins is essential for sensor function in vivo, its precise molecular function is unknown, and no high-resolution crystal structure of any fungal WSC domain is available so far. In the PFAM 35.0 database (state: 11/2021), the CRD/WSC domain (PF01822) can be found in 7660 eukaryotic sequences, 2672 of which are present in a total of 331 different fungal species. Despite their wide distribution, only a few fungal proteins with a WSC domain have been attributed a molecular function. These include the *Sc*Wsc sensors discussed above and a number of fungal orthologs, which also contain a CRD together with a transmembrane domain, and are involved in activation of the CWI pathway [24,25,26,27]. WSC domains have also been found in other domain contexts, e.g., in the β-1-3-exoglucanase *Th*Crd2 of the mycoparasitic fungus *Trichoderma harzianum* [28], and the β-glucan-binding lectin *Si*Wsc3 from the root endophyte *Serendipita indica* [29], indicating a carbohydrate-binding function of CRD/WSC domains. In *Homo sapiens*, the WSC-domain-containing Kremen receptor proteins Krm1/2 have been described to form ternary complexes with the Wnt co-receptors Lrp5/6 and the Wnt antagonistic Dickkopf proteins Dkk1/2 in the vertebrate Wnt/β-catenin signaling pathway [30,31]. Importantly, crystal structures are available for Krm1 (PDB ID 5FWS) and the ternary Krm1–Lrp6–Dkk1 complex (PDB ID 5FWW), which show that the WSC domain of Krm1 forms direct polar contacts with Dkk1 [32]. These studies indicate a second function of CRD/WSC domains in conferring specific protein–protein interactions.

In this study, we aimed at characterizing the structural and functional properties of *Sc*Wsc sensors in more detail. For this purpose, we determined the high-resolution crystal structure of the CRD of *Sc*Wsc1, revealing a tightly packed globular core domain stabilized by four disulfide bonds. The protein surface of the CRD contains three aromatic clusters that are conserved in the CRDs of both *Sc*Wsc2 and *Sc*Wsc3. Our mutational analysis reveals that these surface-exposed aromatic clusters play essential roles in conferring sensor function under cell wall stress conditions, indicating that they might be involved in eliciting appropriate cellular responses via the downstream CWI pathway.

## 2. Materials and Methods

### 2.1. Yeast Strains and Growth Tests

Yeast strain YHUM2959 carrying a *wsc1∆* mutation was obtained by deletion of the *WSC1* gene in yeast strain ESM356-1 (S288c, *MATα*, *ura3-52*, *leu2∆1*, *his3∆200*, *trp1∆63*), [33] following a previously described protocol, and plasmid pFA6a-natNT2 [34].

For growth tests under cell wall stress conditions, yeast strains ESM356-1 or YHUM2959 were freshly transformed with appropriate plasmids (Table 1). Transformants were grown to logarithmic phase in liquid SC-Trp medium, and fivefold serial dilutions of the cultures were spotted onto solid YPD medium supplemented with either 0.4 µg/mL caspofungin (Sigma-Aldrich, Taufkirchen, Germany), 30 µg/mL Congo red (Carl-Roth, Karlsruhe, Germany), 100 µg/mL calcofluor (fluorescent brightener 28, Sigma-Aldrich, Taufkirchen, Germany), or 2 mg/mL caffeine (Sigma-Aldrich, Taufkirchen, Germany). Growth on plates was documented by photography after incubation at 30 °C for 3–5 days. Standard methods for yeast culture medium and transformation were used as described previously [35].

### 2.2. Plasmids

All plasmids used in this study are listed in Table 1. For bacterial production of CRDs from *Sc*Wsc1, *Sc*Wsc2, and *Sc*Wsc3, the pET-28a(+) expression system was used (Merck, Darmstadt, Germany). Construction of expression plasmids BHUM3120, BHUM3121, and BUM3122, respectively, was carried out by the cloning of codon-optimized sequences (BioCat, Heidelberg, Germany) from pMA-based cloning vectors into the expression vector pET-28a(+) by *Nde*I/*Xho*I restriction and ligation. For expression of different *mNeonGreen*-tagged *WSC1* gene variants in *S. cerevisiae*, plasmids BHUM3303, BHMU3304, BHMU3305, BHMU3306, and BHUM3308 were constructed using several steps. (i) In a first step, a 2 kb fragment carrying the *WSC1* locus (−711 to +181 relative to the translational start site ATG at +1) was isolated from the yeast genome by PCR, integrated into the yeast vector pRS314 to obtain plasmid BHUM 3291 (pRS314-WSC1; map shown in Figure S1), and verified by sequencing. (ii) In a second step, plasmids BHUM3293 (pRS314-WSC1^Y22A Y24A Y107A^), BHUM3295 (pRS314-WSC1^Y64A Y70A Y104A^), BHUM3297 (pRS314-WSC1^Y41A W43A Y89A F91A Y93A^), and BHUM3301 (pRS314-WSC1^∆CRD^) were constructed by site-directed mutagenesis using BHUM3291 as a template. All mutations were verified by DNA sequencing. (iii) In a third step, plasmids BHUM 3303 (pRS314-WSC1-mNeonGreen), BHUM3304 (pRS314-WSC1^Y22A Y24A Y107A^-mNeonGreen), BHUM3305 (pRS314-WSC1^Y64A Y70A Y104A^-mNeonGreen), BHUM3306 (pRS314-WSC1^Y41A W43A Y89A F91A Y93A^-mNeonGreen), and BHUM3308 (pRS314-WSC1^∆CRD^-mNeonGreen) were constructed by homologous recombination cloning in yeast using three fragments with overlapping ends. Two of the fragments, which carry the vector pRS314 and the *WSC1* gene or mutated variants, respectively, were obtained by PCR using either one of the plasmids BHUM3291, BHUM3293, BHUM3295, BHUM3297, or BHUM 3301 as templates, and appropriate primers adding 30–40 bp long homologous ends. A third fragment, coding for the *mNeonGreen* gene, was obtained by PCR using plasmid pCR95 [36] as template, and two primers adding appropriate homologous areas. Each three appropriate fragments were then simultaneously transformed into competent yeast cells to obtain the desired plasmids by tripartite homologous recombination. Upon growth of transformed yeast on solid SC-Trp selection medium, resulting plasmids were isolated from transformants, amplified in *E. coli*, and verified by DNA sequence analysis. Maps of the resulting plasmids are shown in Figure S1.

### 2.3. Recombinant Production of CRD Proteins

CRDs of *Sc*Wsc1, *Sc*Wsc2, and *Sc*Wsc3 were produced following a low temperature protocol [38] using the expression plasmids BHUM3120, BHUM3121, and BUM3122, and the *E. coli* strain SHuffle T7 express (New England Biolabs GmbH, Frankfurt, Germany). Proteins were purified by Ni-NTA affinity chromatography (Macherey-Nagel, Düren, Germany) and subsequent size exclusion chromatography using a HiLoad Superdex 75 pg column (GE Healthcare, Munich, Germany). All steps were carried out in AML buffer (20 mM Tris/HCl, pH 8.0, 350 mM NaCl).

### 2.4. CD Spectroscopy and Thermal Shift Assays

CD spectroscopy with recombinant CRDs was performed with a JASCO J-810 spectropolarimeter (JASCO, Pfungstadt, Germany) in quartz cuvettes with a 1 mm gap. Proteins were measured at a concentration of 100 µg/mL in a 50 mM NaHCO_3_ buffer [pH 8.3] in the far UV range (190–260 nm) at 20 °C. All spectra were measured three times against the buffer spectrum. Collected data were converted to molar ellipticity per amino acid. The *Jasco Secondary Structure Estimation* software (JASCO, Pfungstadt, Germany) was used for comparison with a reference spectrum to estimate secondary structure compositions.

Thermal shift assays with recombinant CRDs were performed in a *Rotor-Gene Q* real-time PCR cycler (Qiagen, Hilden, Germany) in volumes of 40 µL, using UV-permeable PCR cups. The temperature was increased linearly by 2 °C per minute. Proteins were measured at a concentration of 1 mg/mL in the presence of *SYPRO*™ Orange dye (7.8×) in either one of the following buffers: 20 mM sodium citrate (pH 3); 20 mM sodium acetate (pH 4); 20 mM sodium acetate (pH 5); 20 mM MES (pH 6); 20 mM HEPES (pH 7); 20 mM Tris-Cl (pH 8); 20 mM CHES (pH 9); 20 mM CHES (pH 10).

### 2.5. Crystallization

Crystal screening with recombinant CRDs was performed with commercially available screens (Qiagen, Hilden, Germany) in a 600 nl sitting drop setup using a Digilab Honeybee 963 dispensing system (Genomic Solutions, Huntingdon Cambridgeshire, UK). Crystals for the CRD of *Sc*Wsc1 belonging to the space group *P*12_1_1 could be obtained at a protein concentration of 70 mg/mL at 18 °C in conditions containing 80 mM sodium acetate (pH 4.5), 1.6 M ammonium sulfate, and 20% (*v*/*v*) glycerol. All crystals were frozen in mother liquor.

### 2.6. X-ray Data Collection, Structure Solution, and Analysis

Datasets were recorded at the ESRF (Grenoble), beamline ID29. Data integration was performed with *XDS* [39]. *XSCALE* [40] or *SCALA* [41] were used for scaling, both run within the CCP4 [42] software suite. The structure of the *Sc*Wsc1 CRD was solved via molecular replacement with *PHASER* [43] using a carefully trimmed model of the WSC domain from the human transmembrane sensor Krm1 (PDB ID 5FWS). Refinement was done with alternating rounds of *phenix.refine* [44] and *Coot* [45].

### 2.7. Western-Blot Analysis

Yeast strains expressing different *mNeonGreen*-tagged *WSC1* gene variants were analyzed by Western blot analysis after growth to logarithmic phase and preparation of protein extracts as previously described [46]. Equal amounts of protein extracts were separated by 12% SDS-PAGE, and transferred to nitrocellulose membranes. Wsc1-mNeonGreen proteins were detected using enhanced chemiluminescence (ECL) technology after incubation of membranes with monoclonal mouse anti-mNeonGreen antibodies (ChromoTek GmbH, Munich, Germany) and peroxidase-coupled goat anti-mouse secondary antibodies (Dianova GmbH, Hamburg, Germany). Membranes were then stripped by treatment with SDS and β-mercaptoethanol, and tubulin was detected by incubation of membranes with monoclonal rabbit anti-tubulin antibodies (Abcam plc, Cambridge, MA, UK) and peroxidase-coupled goat anti-rabbit secondary antibodies (Cayman Chemical, Ann Arbor, MI, USA).

### 2.8. Microscopy

Yeast strains expressing different *mNeonGreen*-tagged *WSC1* gene variants were analyzed by microscopy after growth to logarithmic phase under a Zeiss Axiovert 200M microscope using (i) transmission light microscopy (TM) and (ii) fluorescence microscopy with a GFP filter set (AHF Analysentechnik AG, Tübingen, Germany). Cells were photographed with a Hamamatsu Orca ER digital camera, and pictures were processed and analyzed using the Improvision Volocity software (Improvision, Coventry, UK).

### 2.9. Bioinformatic Analysis

Figures of protein structures were generated with the Molecular Graphics Software PyMOL v2.3.0 (Schrödinger, LLC, New York, NY, USA). Sequence alignments were obtained by using Clustal Omega [47].

### 2.10. Data Availability

The atomic coordinates and structure factors for the *Sc*Wsc1 CRD obtained in this study have been deposited in the Protein Data Bank (www.rcsb.org) under the accession code 7PZ2.

## 3. Results

### 3.1. Characterization of Recombinant CRDs from S. cerevisiae Wsc1, Wsc2, and Wsc3

**Figure 2:**
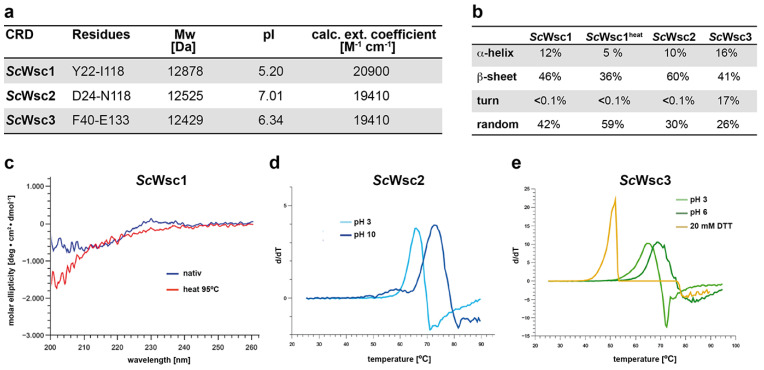
Characterization of recombinant CRDs from *Sc*Wsc1, *Sc*Wsc2, and *Sc*Wsc3. (**a**) Theoretical physiochemical properties of His-tagged proteins. Values were determined with the *ProtParam* tool (https://www.expasy.org; 24 March 2022) using the amino acid sequences encompassing the indicated residues. (**b**) Quantification of secondary structure elements of recombinant CRDs from *Sc*Wsc1, *Sc*Wsc2, and *Sc*Wsc3. Values were determined by CD spectroscopy at 20 °C. For *Sc*Wsc1, values were also determined after heat treatment at 95 °C (heat). (**c**) CD spectroscopy of the recombinant *Sc*Wsc1 CRD before (native; blue line) and after heating to 95 °C (heat; red line) for 1 h. (**d**,**e**) Thermal stability of recombinant CRDs as determined by thermal shift assays (TSA). For *Sc*Wsc2, TSA was performed at pH 3 (light blue line) and pH 10 (dark blue line), respectively (**d**). For *Sc*Wsc3, thermal stability was measured at pH 3 (light green line), pH 6 (dark green line), and at pH 6 in the presence of additional 20 mM DTT (yellow line), respectively (**e**).

In order to biochemically and structurally characterize the CRDs from *S. cerevisiae* Wsc1, Wsc2, and Wsc3, appropriate proteins (Figure 2a) were produced by heterologous expression in *E. coli* and purification employing the hexahistidine (*6xHis*) affinity tag, followed by size-exclusion chromatography. Because removal of the *6xHis* tag profoundly reduced the solubility of the three purified CRDs, further experiments were performed only with the tagged variants. Secondary structure elements of purified proteins were determined by CD spectroscopy, revealing a comparable distribution of α-helical- and β-sheet-containing regions (Figure 2b). In addition, we found a significant stability of the CRD of *Sc*Wsc1 against denaturation by heat treatment up to 95 °C (Figure 2b,c). Further characterization of the recombinant CRDs by thermal shift assays corroborated the high thermal stability of the CRD from *Sc*Wsc1, as no reproducible melting curves could be obtained at different pH values ranging from pH 3 to pH 10 (data not shown). Similarly, no significant melting was observed for the *Sc*Wsc2 and *Sc*Wsc3 CRDs at neutral pH conditions. In the case of *Sc*Wsc2, however, melting curves for the CRD could be obtained either at very acidic conditions with a melting temperature (T_m_) of 66 °C at pH 3, or under alkaline conditions, with a T_m_ of 73 °C at pH 10 (Figure 2d). Similarly, melting was observed for the *Sc*Wsc3 CRD, with melting temperatures of 65 °C at pH 3, and 69 °C at pH 6 (Figure 2e). We also found that addition of the reducing agent DTT significantly decreases the melting temperature of CRDs (Figure 2e, yellow curve), indicating a markedly stabilizing role of the intramolecular disulfide bridges formed by the eight cysteines.

Together, characterization of recombinant CRDs from *Sc*Wsc1, *Sc*Wsc2, and *Sc*Wsc3 reveals a significant pH-dependent stability of WSC domains, and further emphasizes the essential role of their conserved cysteine residues [20,48].

### 3.2. High-Resolution Structure of the ScWsc1 CRD

**Figure 3:**
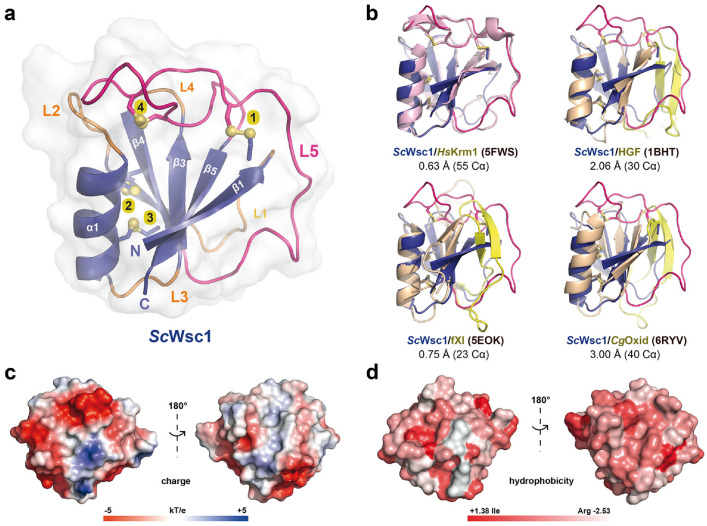
Structural features of the *Sc*Wsc1 cysteine-rich domain. (**a**) The overall structure of the *Sc*Wsc1 CRD (PDB ID 7PZ2; UniProt entry P54867) is shown as a cartoon with the secondary structure elements and the termini indicated. The eight cysteines forming four disulfide bonds (numbered from 1 to 4) are shown as sticks. (**b**) Pairwise superposition between the CRD structures from *Sc*Wsc1 (blue) and human Kremen1 (*Hs*Krm1; PDB ID 5FWS; UniProt entry Q96MU8; light purple), human hepatocytic growth factor (HGF; PDB ID 1BHT; UniProt entry P14210; light orange), human factor XI (fXI; PDB ID 5EOK; UniProt entry P03951; light orange), and a fungal copper oxidase from *Colletotrichum graminearum* (*Cg*Oxid; PDB ID 7PZ2; UniProt entry E3Q9X3; light orange). The PAN/Apple-domain-specific insertions of HGF, fXI, and *Cg*Oxid are highlighted in yellow, the L5 loop of *Sc*Wsc1 in pink. Root mean square standard deviations for the superpositions are indicated in Å, the number of superposed C_α_-positions in parentheses. (**c**) Surface charge and (**d**) surface hydrophobicity predictions for *Sc*Wsc1.

In order to solve the 3D structure of fungal CRDs, and to gain insights into the structural properties of *S. cerevisiae* Wsc family sensors, X-ray diffraction analysis was performed with purified CRD domains. For this purpose, large-scale crystallization screenings were performed with protein concentrations at the highest achievable solubilities. The *Sc*Wsc1 CRD showed a solubility of 70 mg/mL, followed by *Sc*Wsc3 (30 mg/mL) and *Sc*Wsc2 (15 mg/mL). To date, we were only able to obtain crystals of the *Sc*Wsc1 CRD, with first crystals growing after three weeks of incubation. In the cases of *Sc*Wsc2 and *Sc*Wsc3 CRDs, crystallization attempts so far resulted in either proto-crystalline material or precipitation. A crystallographic dataset collected for the *Sc*Wsc1 CRD allowed structure determination by molecular replacement, using as template the structure of the WSC domain from the human transmembrane sensor Krm1 (PDB ID 5FWS) (Figure 3a,b) despite a low sequence identity of only ~20% between the two CRDs. The final structure of the *Sc*Wsc1 CRD was refined at a resolution of 1.6 Å (Table 2).

**Figure 4:**
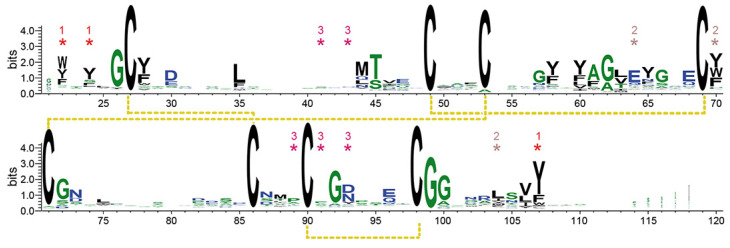
WebLogo [52] generated for 2933 Wsc-type CRD sequences, which have been collected by GREMLIN [53] using the *Sc*Wsc1 sequence A21-S120 as query sequence, and default parameters. Residues belonging to aromatic clusters (Figure 6) have been marked by asterisks. The numbering scheme is derived from *Sc*Wsc1.

The overall structure of the *Sc*Wsc1 CRD is characterized by a central β-sheet, composed of five antiparallel β-strands (β1–β5) which form the core of a tightly packed globular domain (Figure 3a). This β-sheet engulfs an α-helix (α1, S46-G57), and is connected by five loops, L1–L5. Besides the WSC domain of human Krm1, this fold with its β1β5β3β4β2(α1) topology, also corresponds to the PAN/Apple domain fold that has been characterized before for human hepatocytic growth factor HGF [49], human plasma coagulation factor XI [50], and a fungal copper oxidase from *Colletotrichum graminearum* [51] (Figure 3b). The *Sc*Wsc1 CRD is stabilized by the formation of four disulfide bonds from eight highly conserved cysteines (Figure 4), resulting in a highly rigidized structure (Figure 3a). Helix α1 is attached by two disulfide bridges, C49–C69 and C53–C71, to the β4 strand of the β-sheet (Figure 3a; yellow bonds 3 and 4).

**Figure 5:**
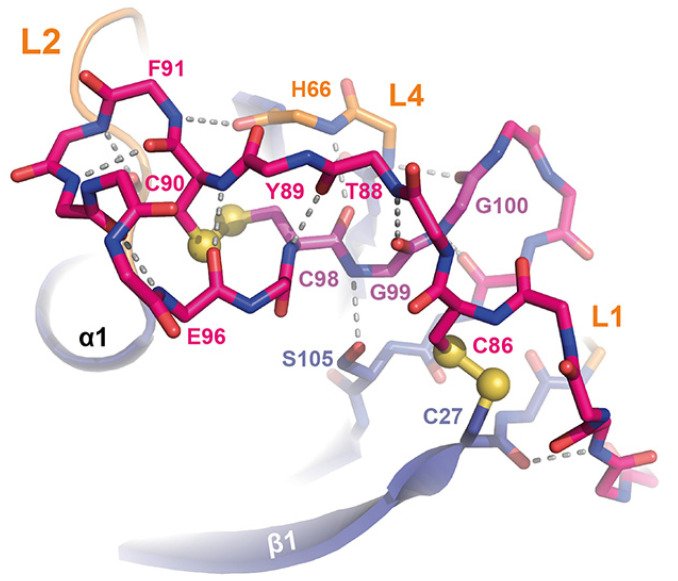
The L5 loop of *Sc*Wsc1. The L5 loop (pink, only main chain shown for S81-A103) covers one end of the PAN/Apple domain together with the accompanying loops L1, L2, and L4. By making numerous interactions, the CGG motif of Wsc-like CRDs (highlighted in purple) is centrally located between the two disulfide bridges C27-C86 and C90-C98, the L4 loop, the N-terminal end of β5, and the crossing L5 stretch N87-C90. Main chain hydrogen bonds of L5 are marked as dashed lines (grey).

Both of these intramolecular cross-links are likewise conserved in the PAN/Apple domains, which have been assigned to a different protein family (PFAM entry PF00024) than the Wsc-like domains (PF01822) due to their low, crosswise sequence similarity. The other two disulfide bridges shown by *Sc*Wsc1 (C27–C86, C90–C98) and the Krm1 CRD are a specific feature of Wsc-like domains due to their strict conservation (Figure 4). These disulfide bridges stabilize the unusually long, but distinctly folded, loop L5 (G72-A103), which enwraps almost the whole PAN/Apple domain (Figure 3a and Figure 5).

Here, one disulfide bridge (Figure 3a; yellow bond 1, C27-C86) connects the β1-strand to the L5 loop, whose conformation is further stabilized by the intra-loop disulfide bridge C90-C98. A comparison of the *Sc*Wsc1 and *Hs*Krm1 CRDs shows that the C-terminal part of this long L5 stretch adopts a conserved conformation, which is stabilized by intra-loop hydrogen bonds, as well as interactions with the L1, L2, and L4 loops (Figure 5). A second determinant for this conformation besides the two disulfide bridges is given by the highly conserved CGG motif of Wsc-like CRDs (*Sc*Wsc1: C98-G100, Figure 4). These two glycines do not only form a hydrogen bonding network from L5 to L4 and β5 (Figure 5), but also allow main chain crossing of L5 between its two conserved disulfide bridges.

**Figure 6:**
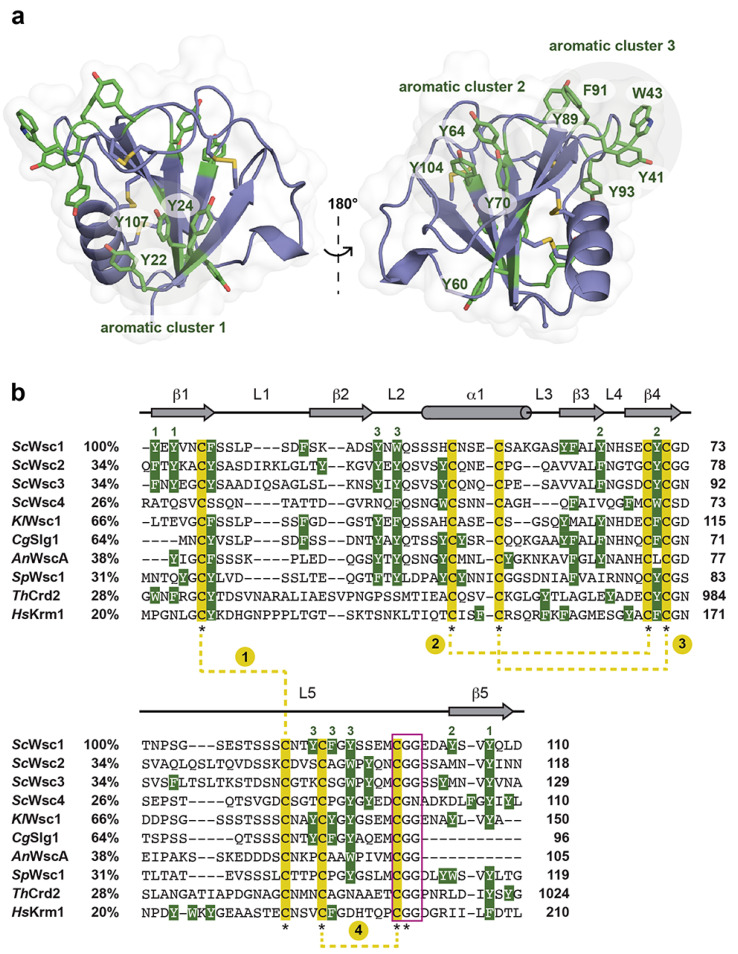
Surface exposure of aromatic residues in fungal CRD/WSC domains. (**a**) Surface-exposed aromatic clusters in the *Sc*Wsc1 CRD. The 12 surface-exposed aromatic residues are shown in green. Three residues form both the aromatic clusters 1 (Y22, Y24, Y107) and 2 (Y64, Y70, Y104), respectively, whereas aromatic cluster 3 encompasses five residues (Y41, W43, Y89, F91, Y93). (**b**) Multiple sequence alignment of selected CRD/WSC domains from *S. cerevisiae* (*Sc*Wsc1, UniProt entry P54867; *Sc*Wsc2, UniProt entry P53832; *Sc*Wsc3, UniProt entry Q12215; *Sc*Wsc4, UniProt entry P38739), *Kluyveromyes lactis* (*Kl*Wsc1, GenBank entry CAH00797.1), *Candida glabrata* (*Cg*Slg1, UniProt entry Q6FUQ7), *Aspergillus nidulans* (*An*WscA, GenBank entry XP_663264.1), *Schizosaccharomyces pombe* (*Sp*Wsc1, UniProt entry P87179), *Trichoderma harzianum* (*Th*Crd2, UniProt entry O14402), and *Homo sapiens* (*Hs*Krm1, UniProt entry Q96MU8). The secondary structure elements of the *Sc*Wsc1 CRD are shown on top (grey). The highly conserved cysteine residues are underlaid in yellow and marked by asterisks. The four disulfide bonds found in the structures of the *Sc*Wsc1 and *Hs*Krm1 CRDs are indicated by yellow dotted lines, and consecutively numbered. Aromatic residues (Phe, Tyr, and Trp) are indicated by white letters, underlaid in green. Green numbers on top indicate the residues of *Sc*Wsc1 forming the three surface-exposed aromatic clusters shown in (**a**). Numbers after protein names indicate percentage identity to *Sc*Wsc1. Numbers on the right indicate position of residues relative to the translational start site. The highly conserved CGG motif is marked by a purple box.

The surface of the Wsc1 CRD lacks an apparent site or pocket for the binding of metal ions or ligands, such as glycans. However, calculation of the electrostatic surface potential reveals a mainly negative charge on one side of the domain, whereas the opposite side has mainly non-polar characteristics (Figure 3c). The negative surface potential results from eleven surface-exposed residues of aspartic and glutamic acids, which explains the acidic pI of 5.20 calculated for the *Sc*Wsc1 CRD (Figure 2a). Calculation of the surface hydrophobicity characteristics does not reveal particularly hydrophobic areas (Figure 3d). However, a further analysis of the surface properties of the *Sc*Wsc1 CRD reveals an unusually high number of surface-exposed aromatic residues (Figure 6), which is reminiscent of the surface-exposed aromatic stretches of yeast adhesins such as Flo11 [54] or Awp1 [55].

**Figure 7:**
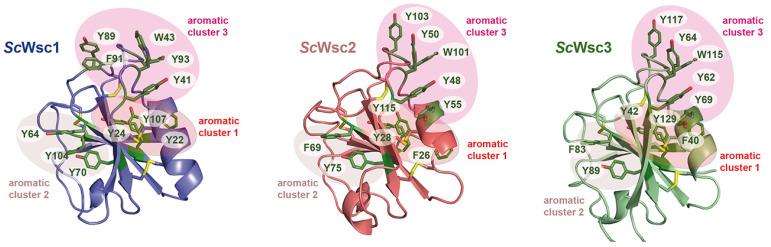
Homology models of *Sc*Wsc2 and *Sc*Wsc3 CRDs. Modelling was performed with *SWISS-MODEL* (https://www.expasy.org; 10 March 2022) based on the crystal structure of the *Sc*Wsc1 CRD (PDB ID 7PZ2) shown on the left. Surface-exposed aromatic residues are shown in green with numbering referring to sequences shown in Figure 5b. Aromatic clusters are indicated by circled areas.

Specifically, twelve of the fifteen aromatic amino acids found in the domain, mostly tyrosines, are surface-exposed, and form three distinct aromatic clusters. The three residues from aromatic cluster 1 (Y22, Y24, Y107) are highly conserved within Wsc-like CRDs, with notable exchanges such as tryptophan or phenylalanine (Figure 4). Likewise, cluster 2 (Y64, Y70, Y104) shows a preference for aromatic residues at least at positions 70 and 104, whereas the larger cluster 3, with its five residues (Y41, W43, Y89, F91, Y93), lacks any apparent conservation pattern (Figure 4 and Figure 6). Nevertheless, a sequence alignment reveals that the CRDs of *Sc*Wsc2 and *Sc*Wsc3 contain aromatic residues at a total of eight positions as well, which correspond to the surface-exposed aromatic sidechains of *Sc*Wsc1 (Figure 6b). Moreover, homology modelling shows that these conserved residues together with two additional non-conserved aromatic residues are also arranged into three aromatic clusters at the CRD surfaces of *Sc*Wsc2 and *Sc*Wsc3 (Figure 7).

In summary, X-ray crystallography of the *Sc*Wsc1 CRD reveals a conserved fold that is characterized by a tightly packed globular core domain enwrapped by the long L5 region, exposing three aromatic clusters which are conserved at least in the CRDs of *Sc*Wsc2 and *Sc*Wsc3.

### 3.3. Functional Characterization of Wsc1 Surface-Exposed Aromatic Clusters

**Figure 8:**
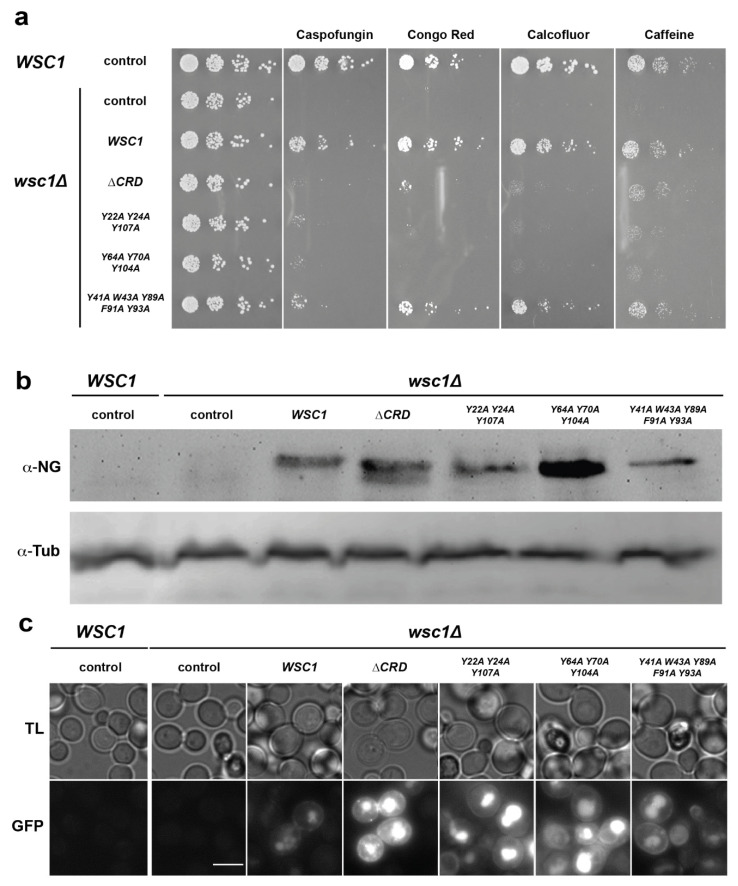
Functional analysis of surface-exposed aromatic residues in the CRD of *Sc*Wsc1. (**a**) A yeast strain carrying a chromosomal deletion of the *WSC1* gene (*wsc1∆*; YHUM2959) was transformed with an empty control plasmid (control; pRS314) or plasmids carrying either WT *WSC1* (BHUM3303) or *WSC1* lacking the CRD (*∆CRD*; BHUM3308) or *WSC1* with mutations in one of the aromatic clusters 1 (Y22A Y24A Y107A; BHUM3304), 2 (Y64A Y70A, Y104A; BHUM3305), or 3 (Y41A W43A Y89A F91A Y93A; BHUM3306). All *WSC1* variants on the plasmids carry a C-terminal translational fusion to the green fluorescent protein mNeonGreen. As an additional control, a yeast strain carrying an untagged chromosomal *WSC1* gene (*WSC1*; ESM356-1) and an empty control plasmid (pRS314) was used (shown on the left). Plasmid carrying strains were grown to logarithmic phase in liquid SC-Trp medium, and fivefold serial dilutions of the cultures were spotted onto solid YPD medium or YPD medium supplemented with either 0.4 µg/mL caspofungin (Caspofungin), 30 µg/mL Congo red (Congo Red), 100 µg/mL Calcofluor white (Calcofluor), or 2 mg/mL caffeine (Caffeine). Plates were photographed after incubation for 3–5 days at 30 °C. The pictures shown are representative for at least two independent transformants. (**b**) Western blot analysis. Strains described in (**a**) were grown to logarithmic phase, and full-length Wsc1-mNeonGreen proteins were detected in cell extracts by anti-mNeonGreen antibodies (α-NG). As an internal loading control, tubulin was detected using anti-tubulin antibodies (α-Tub). The pictures shown are representative for at least two independent transformants. (**c**) Microscopy. All strains were further analyzed for expression of *WSC1* variants by fluorescence microscopy after growth to logarithmic phase using the channels for transmission light (TL) or for mNeonGreen (GFP). The white bar corresponds to 5 µm.

Our finding that CRDs from *S. cerevisiae* Wsc-type mechanosensors carry an unusually high number of surface-exposed aromatic residues prompted us to further analyze their involvement in sensor function, as aromatic clusters have been described to be involved in optimizing both affinity, as well as specificity, of protein–protein and protein–ligand interactions [56,57]. For this purpose, we constructed a set of yeast strains lacking the chromosomal *WSC1* gene (*wsc1∆*), and individually expressing different *WSC1* variants from plasmids. Specifically, we included a WT version, a variant lacking the complete CRD, and three variants each carrying alanine mutations in the residues that form one of the three aromatic clusters. All *WSC1* variants on plasmids were additionally fused to the green fluorescent protein mNeonGreen at their C-terminus, in order to monitor expression levels. Yeast strains were then assayed for viability by serial dilution growth tests in the presence of the β-1,3-glucan synthase inhibitor caspofungin, the chitin-binding agents Congo red and Calcofluor white, and caffeine. As expected, a *wsc1∆* yeast strain carrying a control plasmid without a *WSC1* gene is highly sensitive to the presence of all four agents, whereas strains expressing a genomic or a plasmid borne WT variant of *WSC1* are resistant (Figure 8a).

Expression of the variants either lacking the complete CRD or carrying points mutations in aromatic cluster 1 or 2, respectively, led to enhanced sensitivity towards Caspofungin, Congo red, and Calcofluor white when compared to the strains expressing WT *WSC1*. Somewhat weaker effects were observed on the medium containing caffeine. In contrast, expression of the *WSC1* variant carrying mutations in the aromatic cluster 3 did not confer enhanced sensitivity towards three of the four cell-wall-perturbing agents. These results corroborate the previously described importance of the CRD for sensor function, and reveal an important functional role of surface-exposed aromatic clusters 1 and 2. Importantly, all mutant Wsc1-mNeonGreen fusion proteins were expressed at levels comparable to or above the levels of the WT variant, as detected by Western-blot analysis (Figure 8b). Similarly, GFP signals of mutant Wsc1-mNeonGreen fusion proteins were at levels comparable to or above the levels of the WT variant, as observed by fluorescence microscopy (Figure 8c). However, a noticeable increase of intracellular GFP signals was observed for the variants lacking the CRD or carrying mutations in the aromatic clusters 1 or 2.

In summary, our mutational analysis reveals that the surface-exposed aromatic clusters 1 and 2 play essential roles in conferring *Sc*Wsc1 sensor function under different cell wall stress conditions.

## 4. Discussion

In this study, we have solved the first crystal structure of a fungal cell wall integrity sensor domain: the CRD of *Sc*Wsc1. Together with the WSC domain structure from the human type I transmembrane protein Krm1 [32], our structure shows the typical fold of a PAN/Apple domain that is enwrapped by the long and twisted L5 loop region. This structural determinant apparently replaces the two-stranded antiparallel β-hairpin-like insertions found in the L1 and L5 regions of canonical PAN/Apple domains, such as the human hepatocyte growth factor or coagulation factor XI (Figure 3b). Krm1, together with its paralog Krm2, have been identified as co-receptors for Dickkopf (Dkk) proteins that act as secreted antagonists of vertebrate Wnt-signaling pathways [30]. In the presence of Dkk, Krm paralogs form a ternary complex with the Wnt co-receptors Lrp5/6, leading to endocytosis and amplification of the Wnt-antagonistic activity of Dkk [31]. Reminiscent of the Wsc mechanosensors of *S. cerevisiae*, Krm proteins have a tripartite architecture, and consist of a WSC-domain-containing extracellular region, followed by a transmembrane domain and a short cytoplasmic tail. In contrast to fungal Wsc sensors, however, the extracellular WSC domain of Krm proteins is sandwiched between a Kringle domain (PFAM entry PF00051) and a CUB domain (PFAM entry PF00431). Our study reveals that despite a sequence identity of only 20%, the folds of the *Hs*Krm1 and *Sc*Wsc1 WSC domains are highly similar with their common β1β5β3β4β2(α) topology and stabilization by four disulfide bridges. In addition, both structures show very similar packing arrangements for their L1, L2, L4, and L5 loop regions, and lack an obvious cavity for the binding of ligands. These features are apparently shared by most other known Wsc-like CRD regions, a conclusion that is corroborated by the recently released structural predictions of numerous WSC domains as found in the AlphaFold Protein Structure Database (https://alphafold.ebi.ac.uk). Our study further shows that larger structural differences between the WSC domains of *Hs*Krm1 and *Sc*Wsc1 are mainly local, e.g., within the L3 loop or the N-terminal third of L5, and that the two CRDs have distinct surface properties. Given the low sequence identity of 20%, this finding is not unexpected, but it is noteworthy that the *Sc*Wsc1 CRD carries roughly twice as many surface-exposed aromatic residues than the *Hs*Krm1 WSC domain. One of these aromatic residues of *Hs*Krm1, Y165 (*Sc*Wsc1: S67), has been found to be directly contacting *Hs*Dkk1 [32]. Interestingly, *Hs*Krm1 further contacts *Hs*Dkk1 via a hydrophobic pocket formed by three aromatic residues present on the surface of its Kringle domain, which is adjacent to the WSC domain, and not found in fungal WSC domain sensors. It thus appears that mammalian WSC-type domain receptors have undergone an expansion in their domain architecture and—in comparison to fungal cell wall integrity sensors—exert their sensing functions by employing their CRDs in combination with additional domains.

A central finding of our study is the presence of functionally essential aromatic clusters on the surface of *S. cerevisiae* CRDs. Though we do not yet know the precise function of these aromatic clusters, they could be potentially involved in protein–protein interactions such as the *Hs*Krm proteins [56,57] Our previous work on yeast adhesins demonstrated the crucial role of aromatic stretches on the surfaces of adhesin domains in (i) mediating the formation of cell aggregates by homotypic interactions of neighboring adhesin molecules [54,55], and (ii) conferring kin discrimination [58]. Previous work has also demonstrated that (i) the CRDs of *Sc*Wsc proteins are required for sensor clustering in response to cell wall stress, an event that is thought to trigger the downstream CWI signaling cascade [20]; and (ii) that homodimeric sensor interactions in membrane microdomains (MCMs) depend on CRDs [22]. Thus, aromatic clusters of Wsc CRDs might mediate sensor clustering in MCMs via hydrophobic interactions, and thereby initiate downstream signaling. Our finding that aromatic clusters are present on the CRD surface of three different *Sc*Wsc sensors further indicates that homotypic, as well as heterotypic, sensor interactions are conceivable. This conclusion is supported by the previous demonstration that MCMs formed by *Sc*Wsc1 and *Sc*Wsc2 can overlap [22]. In that regard, CRD-mediated clustering to MCMs resembles models describing cell–cell and cell–substrate interactions mediated by unspecific adhesins such as yeast Flo11 [54,58]. In alternate scenarios, aromatic clusters of Wsc CRDs might of course also mediate bindings to other, yet unknown cell wall components, β-glucans, or proteins, thereby conferring sensor clustering, anchoring in MCMs and/or CWI-pathway-independent structural functions at the yeast cell wall. Finally, our data do not rule out the possibility that aromatic clusters might be involved in intracellular sensor distribution by, e.g., affecting efficiency of their endocytosis (reviewed in [13,17]), and thereby affecting their functionality. A previous study has shown that non-clustering Wsc1 sensors can accumulate in vacuoles [20]. Interestingly, we also observe stronger intracellular GFP signals for some of the non-functional Wsc1 variants (Figure 8c), indicating vacuolar accumulation of such sensors that could be triggered by reduced CRD-mediated clustering.

The CRDs of Wsc sensors have previously been proposed to be involved in binding to glycan structures of the cell wall [20]. However, no direct glycan binding has been experimentally demonstrated so far. Our structural analysis of the *Sc*WSc1 CRD does not reveal an obvious glycan-binding cavity comparable to, e.g., the binding pockets for high-affinity binding of terminal glycans found in fungal adhesins [59,60,61,62,63]. Thus, single WSC domains of fungal mechanosensors alone might not be prone for high-affinity binding of cell wall glycans. However, a recent study has demonstrated that the secreted lectin *Si*Wsc3 (UNiProt entry G4TKP7) from the root endophyte *Serendipita indica* binds to linear long-chain β-1,3-glucans with affinities in the low micromolar range [29]. In contrast to fungal WSC-type sensors, the ~39 kDA integral cell wall component *Si*Wsc3 merely consists of three repeated WSC domains. Interestingly, the individual WSC domains of *Si*Wsc3 also lack an obvious binding pocket for glycan binding, but together appear to form a carbohydrate-binding site [29]. This opens the possibility that high-affinity glycan binding by fungal WSC domain sensors could be enabled by multimerization of individual WSC domains, e.g., upon sensor clustering. Such a mechanism might, for instance, confer anchoring of sensor clusters and MCMs at specific sites of the cell wall, and influence the interaction with downstream signaling components. It might, therefore, be interesting to test whether synthetic multimers of, e.g., *Sc*Wsc domains, are able to efficiently bind to linear long-chain β-1,3-glucans, and if such a binding involves any of the aromatic surface clusters, even though the precise spatial configuration of clustered WSC sensor domains is currently unknown.

Taken together, our study provides novel insights into the structure and function of WSC domains in general, and pinpoints aromatic clusters as functional hotspots of *S. cerevisiae* mechanosensors. As such, our data provide an important basis for future structure-based functional analysis of Wsc-type mechanosensors, and the elucidation of the precise role of CRDs in sensor clustering, downstream signaling, and interactions with other cell wall proteins and/or glycans.

## Figures and Tables

**Table 1:** Plasmids used in this study.

Plasmid	Relevant Genotype	Source
pET-28(a)+	*P_T7_-6xHis lacI Kan^R^*	Merck, Germany
BHUM3120	*WSC1^(22−118)^* in pET-28(a)+	this study
BHUM3121	*WSC2^(24−118)^* in pET-28(a)+	this study
BHUM3122	*WSC3^(40−133)^* in pET-28(a)+	this study
pFA6a-natNT2	*PFA6a-NatNT2 Amp^R^*	[34]
pCR95	*mNeonGreen::NatNT2 Amp^R^*	[36]
pRS314	*TRP1 ARS CEN2 Amp^R^*	[37]
BHUM3291	*WSC1* in pRS314	this study
BHUM3293	*WSC1^Y22A Y24A Y107A^* in pRS314	this study
BHUM3295	*WSC1^Y64A Y70A Y104A^* in pRS314	this study
BHUM3297	*WSC1^Y41A W43A Y89A F91A Y93A^* in pRS314	this study
BHUM3301	*WSC1^∆CRD^* in pRS314	this study
BHUM3303	*WSC1-mNeonGreen* in pRS314	this study
BHUM3304	*WSC1^Y22A Y24A Y107A^*-mNeonGreen in pRS314	this study
BHUM3305	*WSC1^Y64A Y70A Y104A^-mNeonGreen* in pRS314	this study
BHUM3306	*WSC1^Y41A W43A Y89A F91A Y93A^-mNeonGreen* in pRS314	this study
BHUM3308	*WSC1^∆CRD^-mNeonGreen* in pRS314	this study

**Table 2:** Data collection and refinement statistics of *Sc*Wsc1^CRD^.

PDB Code	7PZ2
X-ray source	ESRF ID29
Wavelength (Å)	0.979
Resolution range (Å) ^1^	26.75–1.58 (1.64–1.58)
Space group	*P* 1 2_1_ 1
Unit cell	*a* = 31.82 Å, *b* = 53.5 Å, *c* = 51.93 Å *α* = 90°, *β* = 95.46°, *γ* = 90°
Total reflections ^1^	70,058 (6136)
Unique reflections ^1^	23,125 (2133)
Multiplicity ^1^	3.0 (2.8)
Completeness (%) ^1^	97.06 (91.42)
Mean I/sigma(I) ^1^	10.35 (2.17)
Wilson *B*-factor (Å^2^)	16.72
*R*_merge_ ^1^	0.06339 (0.4653)
CC_1/2_ ^1^	0.997 (0.756)
CC* ^1^	0.999 (0.928)
Reflections used in refinement ^1^	23,052 (2132)
Reflections used for *R*_free_ ^1^	1113 (126)
*R*_work_ ^1^	0.150 (0.258)
*R*_free_ ^1^	0.184 (0.251)
CC (work, free) ^1^	0.973 (0.819), 0.965 (0.824)
Number of non-hydrogen atoms	1731
Macromolecules	1527
Ligands, solvent	7197
Protein residues	197
r.m.s.d. bonds (Å)	0.005
r.m.s.d. angles (°)	0.99
Ramachandran favored (%)	92.23
Ramachandran allowed (%)	7.77
Ramachandran outliers (%)	0.00
Rotamer outliers (%)	0.56
Clashscore ^2^	1.06
Average B-factor (Å^2^)	20.88
Macromolecules (Å^2^)	19.53
Ligands, Solvent (Å^2^)	25.84, 31.15

^1^ Values in parentheses correspond to highest resolution shell. ^2^ As calculated by MolProbity.

## Data Availability

Original datasets, strains, and constructs employed in this work are freely available upon request to the academic community for research purposes only.

## Supplementary Materials

The following are available online at https://www.mdpi.com/article/10.3390/jof8040379/s1, Figure S1: Plasmid maps.
