# Supplementary material for: Structure of the Yeast Cell Wall Integrity Sensor Wsc1 Reveals an Essential Role of Surface-Exposed Aromatic Clusters
Source: J Fungi (Basel). 2022 Apr 8;8(4):379. doi: 10.3390/jof8040379 (PMC9024836; doi:10.3390/jof8040379)
Supplement: Supplementary file 1 [file jof-1674496-supplementary.pdf › jof-1674496-supplementary.pdf]

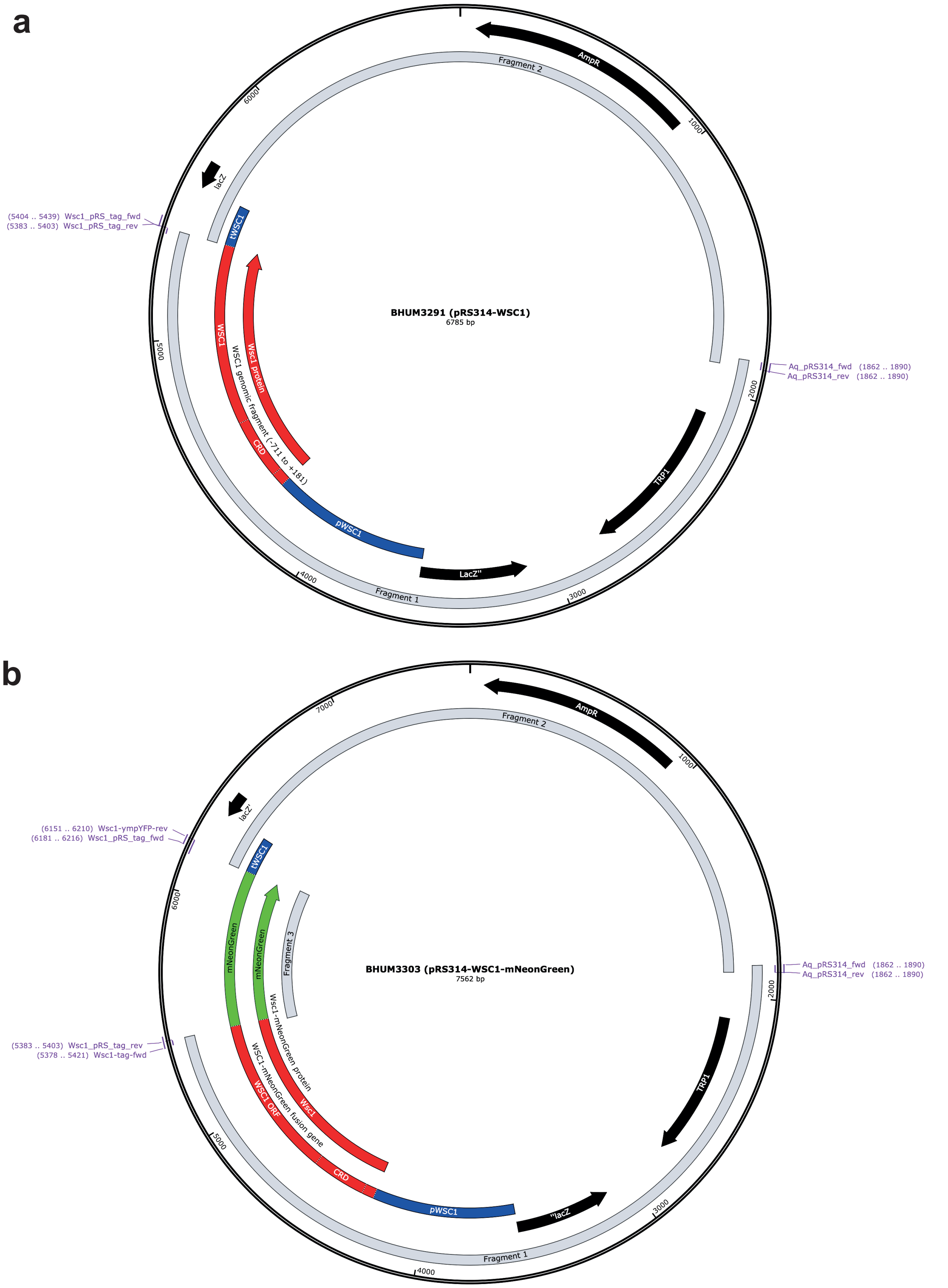

Figure S1. Plasmid maps. (**a**) Map for plasmid BHUM3291 (pRS314-WSC1). The 2 kb *WSC1* genomic fragment (-711 to +181 relative to the translational ATG start site at +1) encompasses the *WSC1* ORF region (WSC1; red), the *WSC1* promoter region (pWSC1; 711 bp; blue;) and the *WSC1* terminator region (tWSC1; 181 bp; blue). The CRD region of the *WSC1* ORF is indicated. Elements of the centromere-based vector pRS314 (*AmpR*, *TRP1* and *lacZ*) are indicated in black. The encoded Wsc1 protein in indicated by an arrow. Fragments 1 and 2, which were used to construct plasmid BHUM3303 depicted in (b), are shown in grey and were generated by PCR using BHUM3291 as template and the four primers indicated in magenta. Maps for plasmids BHUM3293 (pRS314-WSC1$^{Y22A\ Y24A\ Y107A}$), BHUM3295 (pRS314-WSC1$^{Y64A\ Y70A\ Y104A}$), BHUM3297 (pRS314-WSC1$^{Y41A\ W43A\ Y89A\ F91A\ Y93A}$) and BHUM3301 (pRS314-WSC1$^{\Delta CRD}$), which carry mutations in the CRD of *WSC1* and that were constructed by site directed mutagenesis using BHUM3291 as template, are identical (except for mutations). (**b**) Map for BHUM3303 (pRS314-WSC1-mNeonGreen). BHUM3303 was generated by tripartite *in vivo* recombination of three overlapping fragments (Fragments 1 to 3; grey). Fragments 1 and 2, carrying the *WSC1* genomic region and vector pRS314, were generated as described in (a). Fragment 3, carrying the *mNeonGreen* ORF (shown in green), was obtained by PCR using plasmid pCR95 [36] and the two primers Wsc1-tag-fwd and Wsc1-ympYFP-rev (magenta). The encoded Wsc1-mNeonGreen fusion protein is indicated by an arrow. Plasmids BHUM3304 (pRS314-WSC1$^{Y22A\ Y24A\ Y107A}$-mNeonGreen), BHUM3305 (pRS314-WSC1$^{Y64A\ Y70A\ Y104A}$-mNeonGreen), BHUM3306 (pRS314-WSC1$^{Y41A\ W43A\ Y89A\ F91A\ Y93A}$-mNeonGreen) and BHUM3308 (pRS314-WSC1$^{\Delta CRD}$-mNeonGreen), which carry mutations in the CRD of *WSC1*, were constructed following the same strategy, but by using instead of BHUM 3291 either of the plasmids BHUM3293, BHUM3295, BHUM3297 or BHUM3301 as templates to obtain Fragments 1 and 2. Therefore, these plasmids have maps identical to BHUM3303 (except for mutations).
